# The Impact of Homocysteine on the Risk of Hormone-Related Cancers: A Mendelian Randomization Study

**DOI:** 10.3389/fnut.2021.645371

**Published:** 2021-08-24

**Authors:** Qian He, Ze Yang, Yandi Sun, Zihao Qu, Xueyao Jia, Jingjia Li, Yindan Lin, Yan Luo

**Affiliations:** ^1^Department of Biochemistry and Cancer Institute of the Second Affiliated Hospital, Zhejiang University School of Medicine, Hangzhou, China; ^2^Key Laboratory of Cancer Prevention and Intervention of China National Ministry of Education, Hangzhou, China; ^3^Department of Orthopedic Surgery, The Second Affiliated Hospital, Zhejiang University School of Medicine, Hangzhou, China

**Keywords:** homocysteine, breast cancer, prostate cancer, renal cell carcinoma, Mendelian randomization

## Abstract

**Background:** Aberrant homocysteine level is associated with metabolic disorders and DNA damage, which may be involved in the carcinogenesis of hormone-related cancers, but clinical results of observational studies are controversial. In this study, we investigated the causal relationships between plasma homocysteine and breast cancer (BRCA), prostate cancer (PrCa), and renal cell carcinoma (RCC) using Mendelian randomization (MR) analyses.

**Design and Methods:** To investigate the putative causal associations between homocysteine and the aforementioned three types of cancers, a two-sample MR study was employed for the study. The primary strategy for summary data analyses was the inverse-variance-weighted (IVW) approach. In our study, the single-nucleotide polymorphisms (SNPs) excluded confounding factors through Linkage Disequilibrium (LD). Phenoscanner tests were the instrumental variants (IVs), homocysteine was the exposure, and BRCA, PrCa, and RCC were the outcomes. Single-nucleotide polymorphisms associated with homocysteine were extracted from a large genome-wide association study (GWAS) meta-analysis of European participants (*n* = 44,147). Summary Statistics of BRCA were obtained from the latest and largest GWAS meta-analysis comprising of 82 studies from Breast Cancer Association Consortium (BCAC) studies, including women of European ancestry (133,384 cases and 113,789 controls); we obtained summary-level data from the GWAS meta-analysis of PrCa comprising 79,148 cases and 61,106 controls of European ancestry, and the dataset of RCC was a sex-specific GWAS meta-analysis comprising of two kidney cancer genome-wide scans for men (3,227 cases and 4,916 controls) and women (1,992 cases and 3,095 controls) of European ancestry. The MR-Egger and weight median analyses were applied for the pleiotropy test.

**Results:** The results showed null associations between plasma homocysteine levels and overall BRCA (effect = 0.97, 95% CI: 0.90–1.06, *P* = 0.543), overall PrCa (effect = 1.01, 95% CI: 0.93–1.11, *P* = 0.774), RCC in men (effect = 0.99, 95% CI: 0.73–1.34, *P* = 0.929), and RCC in women (effect = 0.89, 95% CI: 0.61–1.31, *P* = 0.563).

**Conclusions:** We found no putative causal associations between homocysteine and risk of BRCA, PrCa, and RCC.

## Introduction

Homocysteine is derived from the essential amino acid methionine (1) and functions as a metabolic intermediate of the methionine cycle that is essential for one-carbon metabolism (2). Activated by ATP, methionine is converted to S-adenosyl methionine (SAM), a donor for DNA methylation (3) that also leads to producing homocysteine as a crucial intermediate for methionine regeneration. This process requires a 5-methyl-tetra-hydrogen-folate (5-MTHF) unit and a specialized methyltransferase that uses vitamin B12 as a coenzyme (4). Hence, deficiency of folic acid and vitamin B12 may lead to a defective methionine cycle thus the accumulation of the plasma homocysteine level (5, 6), which has been linked to an elevated risk of cardiovascular and metabolic disorders (7–9). Indeed, plasma levels of these one-carbon-unit related metabolites are usually measured for clinical diagnosis of cardiovascular diseases and atherosclerosis. Conversely, reduced plasma homocysteine levels had been reported in some clinical cases of patients who received hormone replacement therapy for post-menopause or severe hypothyroidism (10, 11). On one hand, this negative correlation between hormone and homocysteine levels may be explained by the effects of hormones (estrogen or thyroid hormones) on various enzymes including those involved in methionine metabolism. On the other hand, homocysteine levels have been found to be elevated in the plasma of patients with breast cancer (BRCA), colorectal cancer, primary hepatocellular carcinoma, and many other malignancies (12, 13); this might be manifested by altered methionine cycle that is robust during cancer pathogenesis and is related to facilitated DNA double strand breaks and other mutations. Indeed, elevated homocysteine levels have been linked to tumorigenesis through DNA hypomethylation and inactivation of tumor suppressor genes (14, 15), and it has been noted for some years that plasma homocysteine concentrations are associated with risks of tumorigenesis (16–18). However, the causal relationship between homocysteine and cancers remains controversial. For instance, certain observational studies suggest that homocysteine is a risk factor for some cancers, e.g., BRCA and prostate cancer (PrCa) (19, 20). Other studies, however, suggest otherwise (21–24).

Breast cancer constitutes a malignancy with the highest incidence rate in women worldwide (25). Epidemiological investigations showed that high estrogen levels can significantly change the endocrine environment *in vivo* and induce the carcinogenesis of BRCA (26, 27). Estrogen triggers transcriptional programs through estrogen receptors and cognate co-activators, and target genes include certain oncogenes that are deregulated in BRCA cells—which are thought to be the primary driving forces for the pathogenesis (28). The wealth of evidence shows that the high incidence of BRCA and ovarian cancer in women (29), along with PrCa and testicular cancer in men, might be related to the disordered hormonal status, presumably owing to the fact that relevant tissues are sensitive to hormones and chemicals that disrupt hormone balance. For instance, serum androgen level is one of the important indicators in the initial diagnosis of the majority of PrCa (30, 31), and clinical endocrine therapy of PrCa is mainly to reduce androgen levels and to inhibit the function of the androgen receptor (32). Renal cell carcinoma may also be hormone-related cancer, given that clinical studies have found that multiple hormone receptors were highly expressed in RCC tumor tissues (33) and abnormal hormone levels are of vital importance for RCC development (34, 35). In a sense, BRCA, PrCa, and RCC are all hormone-related cancers.

Epidemiological/observational studies have limitations because of (potentially) biased results, limited cases, confounding factors, and reverse causation. It is interesting to note that published observational studies on the association of homocysteine, as a potential cancer biomarker, with risks of BRCA and PrCa have drawn inconsistent conclusions (19–24). One limitation of these studies is that it is difficult to interpret the results with confounding and reverse causation. Mendelian randomization (MR) study design has significant strengths by using randomlyassigned SNPs as proxies for exposure to evaluate its causal effects on the outcome in the absence of pleiotropic effects and reverse causality (36, 37), which is unachievable by epidemiological/observational studies. Whether excess homocysteine is the cause of cancer without confounding factors remains unclear. Therefore, we designed a two-sample MR analyses to solve the problem. The MR analyses using genome-wide association study (GWAS) data have been widely employed to establish casual relationships between (genetic) exposure factors and clinical outcomes. According to the genetic law of Mendel's, SNPs are randomly-assigned during gamete formation, which can evaluate the causality between an exposure and an outcome. Mendelian randomization analyses need to satisfy three assumptions: (1) selected instrumental variant (IV) must be associated with the exposure factor; (2) selected IV can only have an effect on the outcome through the exposure; and (3) selected IV cannot be affected by the confounding factors that have an impact upon the association between the exposure and the outcome. In our study, the SNPs excluded confounding factors were the IVs, homocysteine is the exposure, and BRCA, PrCa, and RCC are the outcomes. This study aimed to identify putative causal associations between homocysteine and risks of BRCA, PrCa, and RCC.

## Materials and Methods

### Genetic Associations With the Plasma Level of Homocysteine

**Figure 1:**
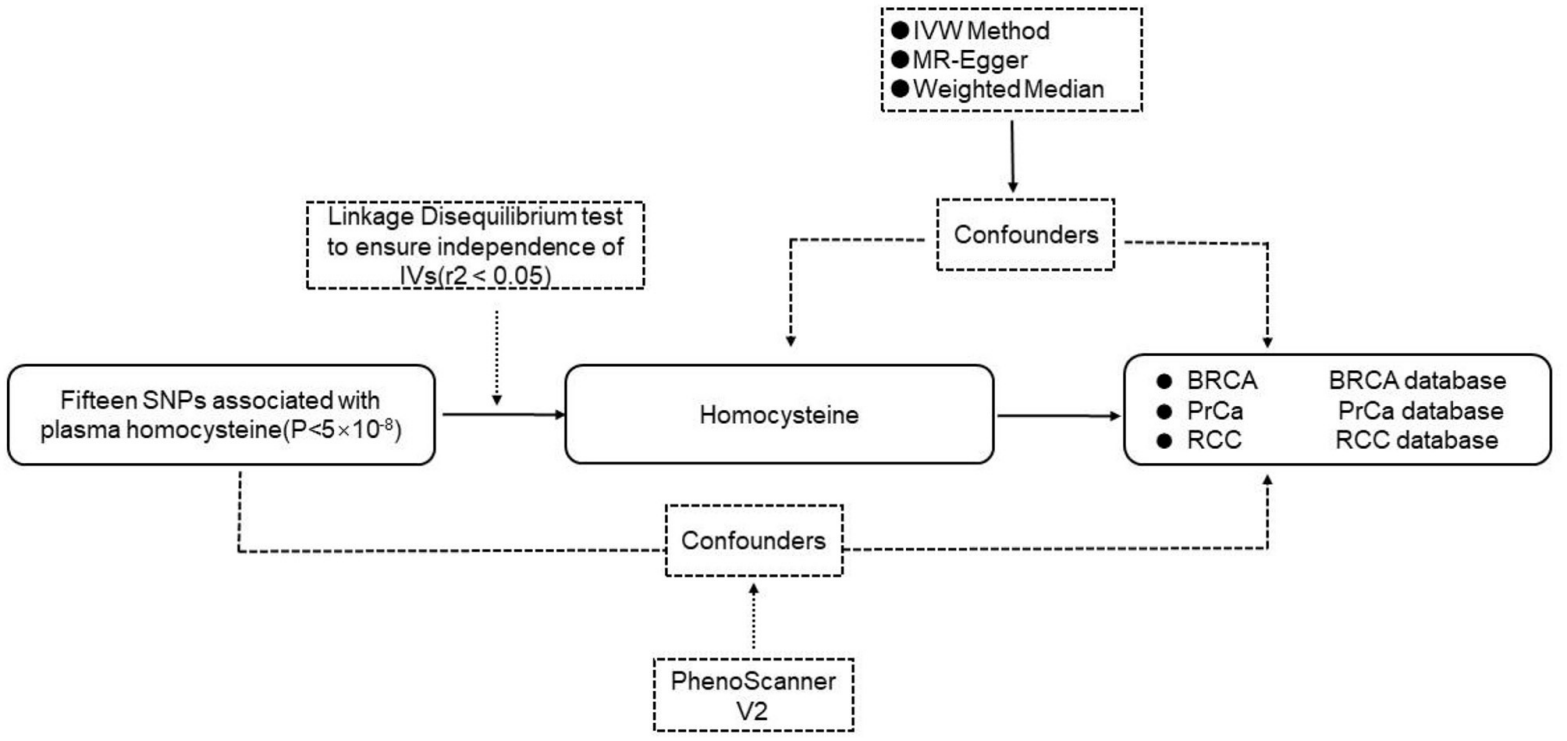
The flow chart of the MR study. IVs, instrumental variables; SNP, single nucleotide polymorphism; IVW, inverse-variance-weighted; BRCA, breast cancer; PrCa, prostate cancer; RCC, renal cell carcinoma.

The flow chart of the MR study design is shown in Figure 1. We designed the MR analyses based on the aforementioned three assumptions to assess the associations between homocysteine and risks of BRCA, PrCa, and RCC.

Candidate IVs linked to plasma homocysteine concentrations were selected from a study that included data from 44,147 participants of European ancestry on total plasma homocysteine concentrations (38). This meta-analysis included individual-level data from 10 cohorts. Plasma homocysteine levels were measured in each cohort by Liquid chromatography–tandem mass spectrometry, high performance liquid chromatography, and enzymatic methods. The participants from each cohort were genotyped by using the Affymetrix 5.0 GeneChip 500K 500K (Thermo Fisher), Illumina Infinium HumanHap610K, 550, and Illumina HumanCNV370 BeadChips (Illumina). The exclusion criteria for SNPs in each cohort [minor allele frequency (MAF) <1%, *P*-value for the Hardy–Weinberg equilibrium (HWE) <1.0 × 10^−6^, call rate <95%] were used. General characteristics of the contributing cohorts and study population are described elsewhere (38). Briefly, the age of the participants of each cohort ranged from 17 to 79 years. Except for the four cohorts [The TwinsUK cohort (TUK1&TUK2), Women's Genome Health Study (WGHS), and Nurses' Health Study (NHS)], which are mainly composed of women, the number of men and the number of women in the rest of the cohorts are roughly the same (women: 45–58%).

### IVs Selection and Validation

To single out the genetic variables linked to plasma homocysteine concentrations, a genome-wide significance (*P* < 5 × 10^−8^) was set as the standard to ensure a close relationship between SNPs and plasma homocysteine levels. A total of 18 SNPs were obtained from a study of total homocysteine level datasets. Among them, three SNPs (rs1801133, rs957140, and rs12921383) were weeded out because of the linkage disequilibrium (LD) to conform the independence of selected IVs (*r*^2^ < 0.05) (Supplementary Table 1). Given the primary assumption of MR analyses that IVs can only affect the outcome through the exposure, we also performed a search on the Phenoscanner website to detect the pleiotropic effects of the selected IVs and removed one SNP (rs12921383) due to its link to skin cancer; in fact, this one overlapped with the aforementioned three SNPs. Eventually, the remaining 15 SNPs were selected to analyze the relationships between genetic variants for homocysteine levels and the risk of BRCA. The characteristics of the 15 SNPs are shown in Table 1 and Supplementary Table 2.

**Table 1:** The characteristics of 15 valid SNPs and their associations with homocysteine levels.

**SNP**	**Nearby gene**	**EA**	**Explained variant (%)**	**Chr**	**Eeffect allele**	**Association with homocysteine**		
						**Beta**	**SE**	***P***
rs2275565	MTR	0.79	0.1	1	T	−0.0542	0.009	1.96E-43
rs4660306	MMACHC	0.33	0.08	1	T	0.0435	0.007	2.33E-12
rs12134663	MTHFR	0.2	0.33	1	A	−0.101	0.011	2.54E-09
rs1047891	CPS1	0.33	0.33	2	A	0.0864	0.008	4.58E-27
rs9369898	MUT	0.62	0.1	6	A	0.0449	0.007	2.17E-24
rs548987	SLC17A3	0.13	0.08	6	C	0.0597	0.01	1.12E-11
rs42648	GTPB10	0.6	0.07	7	A	−0.0395	0.007	1.97E-10
rs1801222	CUBN	0.34	0.09	10	A	0.0453	0.007	8.43E-10
rs12780845	CUBN	0.65	0.13	10	A	0.0529	0.009	7.8E-09
rs7130284	NOX4	0.93	0.2	11	T	−0.1242	0.013	1.88E-21
rs2251468	HNF1A	0.35	0.12	12	A	−0.0512	0.007	1.28E-10
rs154657	DPEP1	0.47	0.46	16	A	0.0963	0.007	1.74E-20
rs838133	FUT2	0.45	0.09	19	A	0.0422	0.007	7.48E-10
rs234709	CBS	0.55	0.26	21	T	−0.0718	0.007	3.9E-12
rs2851391	CBS	0.47	0.16	21	T	0.056	0.008	0.000000017

*SNP, single nucleotide polymorphism; EAF, frequency of efect allele; Chr, chromosome; SE, standard error*.

### Study Outcomes

Breast cancer, PrCa, and RCC were the outcomes. We selected the latest published GWAS meta-analysis that contained the most complete clinical data available, on three types of cancers, respectively. The sources of datasets are shown in Table 2.

**Table 2:** The characteristics of genome-wide association studies (GWAS) on the included outcomes.

**Outcome**	**SNPs**	**Consortium**	**Total population**	**Cases/Controls**	**Ethnicity**	**References**
Overall BRCA	15	BCAC	267,173	133,384/133,789	European	Genome-wide association study identifies 32 novel breast cancer susceptibility loci from overall and subtype-specific analyses. Pubmed ID:32424353
Overall PrCa	15	PRATICAL	140,254	79,148/61,106	European	Association analyses of more than 140,000 men identify 63 new prostate cancer susceptibility loci. Pubmed ID:29892016
RCC in men	15	IARC	8,143	3,227/4,916	European	Sex specific associations in genome wide association analysis of renal cell carcinoma. Pubmed ID:31231134
RCC in women			5,087	1,992/3,095		

*Single-nucleotide polymorphisms (SNPs) represent the number of SNPs selected as instrumental variables for plasma levels in analysis with each outcome*.

Summary statistics of overall BRCA were obtained from the latest and largest GWAS meta-analysis comprising of 82 studies from the Breast Cancer Association Consortium (BCAC), including women of European ancestry (133,384 cases and 113,789 controls) [BRCA–GWAS] (39). Two Illumina iSelect genotyping arrays, iCOGS and OncoArray, were applied for sample genotyping in this study. Single-nucleotide polymorphisms with MAF < 0.005 were excluded.

For PrCa, we extracted summary-level data from the GWAS meta-analysis of Prostate Cancer Association Group to Investigate Cancer-Associated Alterations in the Genome (PRATICAL) consortium comprising of 79,148 cases and 61,106 disease-free controls of European ancestry [PrCa–GWAS] (40). This GWAS meta-analysis included the PrCa GWAS meta-analysis published in 2014, and the summary data of PrCa samples from 52 studies genotyping on the OncoArray analysis published in 2017. The QC criteria excluded SNPs with *P*-value for HWE < 1.0 × 10^−12^ in cases or *P* < 10^−7^ in controls, the call rate <95%, or with concordance <98%.

The dataset of RCC was a sex-specific GWAS meta-analysis comprising of two kidney cancer genome-wide scans for men (3,227 cases and 4,916 controls) and women (1,992 cases and 3,095 controls) of European ancestry [RCC–GWAS] (41). HumanHap 317k, 550, or 610Q were conducted to genotype the selected participants. Standard QC procedures (*P*-value for HWE <1.0 ×10–7, call rate <90%, and MAF < 0.05) were applied.

### Statistical Analyses

In our study, three different statistical methods of two-sample MR analyses were employed. First of all, the inverse-variance-weight (IVW) approach (42) was applied for the primary two-sample MR analyses to quantify the causal associations between plasma homocysteine concentrations and the risk of three types of cancers. Briefly, the ratio of coefficients was calculated in the IV outcome regression to evaluate the causal effects. ME-Egger regression approach was used to examine the horizontal pleiotropy between IVs and the three types of cancers, independent of homocysteine levels. The intercept differs from zero (*p* > 0.05) and indicates no horizontal pleiotropy. In addition, a weighted median method (WM), an effective statistical tool that only needs half of the effective SNPs, was used as proof for the IVW approach by calculating the median value of selected estimates of IVs (43).

All analyses were conducted by “Mendelian Randomization” package in R version 3.6.1.

## Results

### Causal Associations With Diverse Cancers

To establish putative causal associations between homocysteine concentrations and BRCA, PrCa, and RCC, we performed IVW analyses to estimate causal effects. The associations between genetic variants for plasma concentrations of homocysteine and all the outcomes are listed in Supplementary Tables 3–5. Our study suggested null significant causal associations between homocysteine concentrations and three types of cancers.

**Figure 2:**
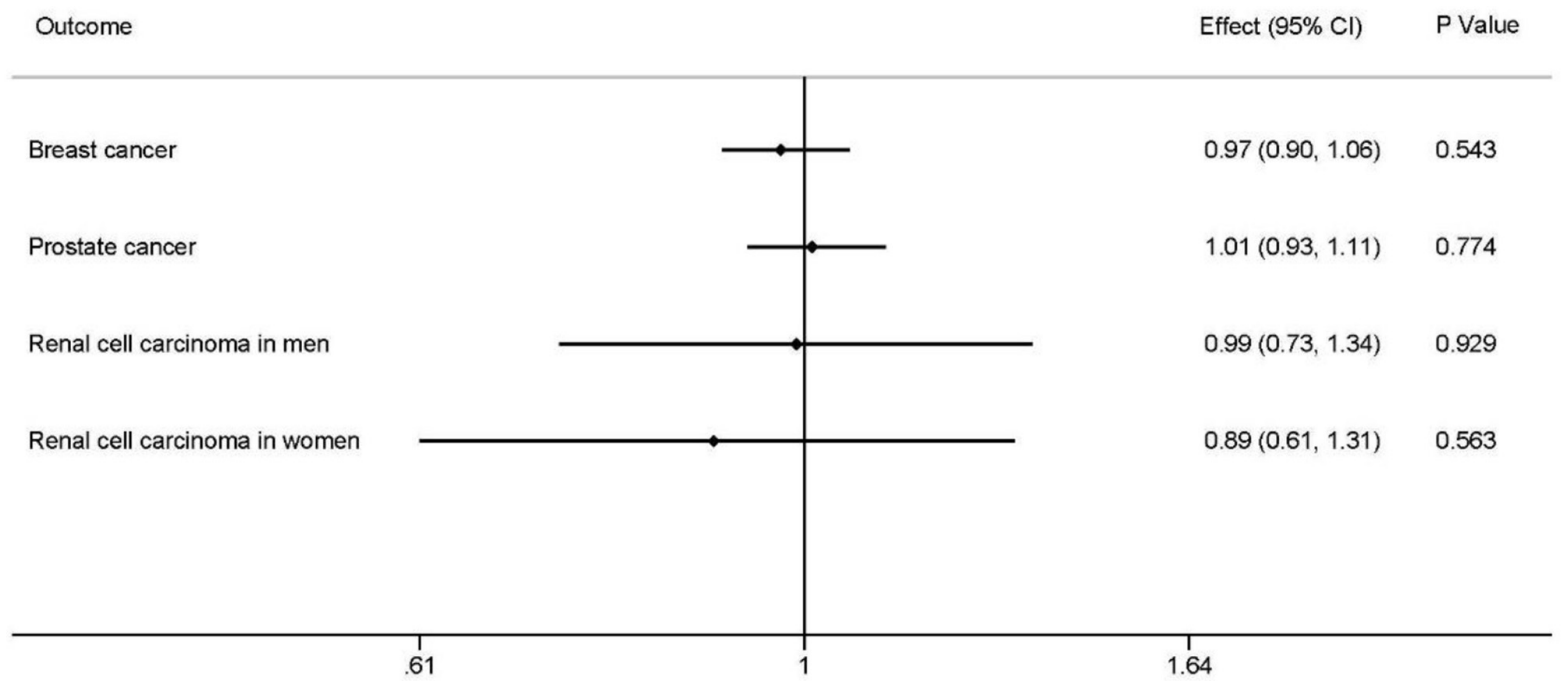
Causal associations between homocysteine levels and overall breast cancer, overall prostate cancer, renal cell carcinoma in men, and renal carcinoma in women. The effects, 95% CIs, and *P*-values of associations are contained. Effect, the combined causal effect; CI, confidence interval; *P*-value, *P*-value of the causal estimate.

The outcomes suggested no evidence that homocysteine levels had causal effects on overall BRCA (effect = 0.97, 95% CI: 0.90–1.06, *P* = 0.543; Figure 2; Supplementary Table 6).

There was no significant relationship between homocysteine levels and overall PrCa (effect = 1.01, 95% CI: 0.93–1.11, *P* = 0.774; Figure 2; Supplementary Table 6).

Inverse-variance-weight analyses showed no effects of homocysteine concentrations on RCC of different sexes, RCC in men (effect = 0.99, 95% CI: 0.73–1.34, *P* = 0.929), and RCC in women (effect = 0.89, 95% CIl: 0.61–1.31, *P* = 0.563) (Figure 2; Supplementary Table 6).

### Sensitivity Analyses

The MR-Egger and the weight medium methods were used to further analyze the influence of pleiotropic effects on the final outcome. A similar conclusion was drawn according to the sensitivity analyses. The MR-Egger test showed non-zero intercept values (*p* > 0.05), which indicated little horizontal pleiotropy (Supplementary Table 7). Similar results were observed in the WM analyses (Supplementary Table 7). All the results confirmed no pleiotropy effects on our estimated associations.

## Discussion

This two-sample MR study suggested no putative relationships between plasma homocysteine levels and the risk of BRCA, PrCa, and RCC. In addition, the levels of homocysteine were not significantly related to the risk of BRCAs with different ER expression status and RCC of different sexes.

As a plasma component involved in one-carbon metabolism, homocysteine has attracted attention in diverse clinical studies. Higher plasma homocysteine levels tend to be associated with metabolic disorders, even cancer; but the conclusions drawn from previous observational studies on associations between levels of plasma homocysteine with cancer risk are controversial given that other studies found no evidence for casual associations between genetically-determined plasma homocysteine levels with risk of hormone-related cancers. In an EPIC–Varese cohort study (23), researchers recruited 276 BRCA cases to study on the role that one-carbon metabolism plays in breast carcinogenesis. The team concluded that high homocysteine levels were not linked to BRCA risks; however, others found that increased homocysteine levels were inversely related to multiple cancers (17–19). Some case control studies also found that SNPs existed in genes involved in homocysteine metabolism. These polymorphisms were reported to be closely related to different cancers. These controversial findings may be explained by the following reasons: (1) The sample size of these observational studies might not be large enough to draw a valid conclusion; (2) establishing causality is a much more difficult task which many epidemiological techniques cannot achieve; (3) observational studies are prone to residual confounding factors, such as diversified nutrients involved in one-carbon metabolism, which results in biased conclusion, and thus inaccuracy. It is still unknown whether homocysteine is one of the causes of cancers or the result. Therefore, our MR analyses wanted to explore whether homocysteine is the cause of cancer without confounding factors. Our findings that plasma homocysteine levels had no causal effect on the risk of cancer are consistent with the EPIC–Varese cohort study.

The plasma homocysteine levels were not found to be significantly elevated in early stage cancers, as opposed to a sharp increase in their advanced stages (17). In addition, pre-menopausal and post-menopausal patients with BRCA showed different levels of plasma homocysteine (44). Moreover, upon patients receiving surgery or chemotherapy, their plasma homocysteine levels increased significantly leading to a higher incidence of thromboembolism (45), one of the most frequent complications caused by hyperhomocysteinemia (46). Regarding why homocysteine concentrations differ among different stages of cancer development whereas they lack association with cancer risk, we speculate that homocysteine might not be an independent risk factor, that is, it exerts effects by interplaying with other exposure factors. At an early stage of cancers, the homeostatic level of homocysteine may suffice to support cancer cell growth in conjunction with other methionine cycle components such as 5-MTHF, the methyl-group carrier, or other metabolite(s) like folate. An observational study suggested that women with higher homocysteine levels and low folate levels may be at risk of increased BRCA (16). It was indeed suggested that one or more micronutrients involved in homocysteine metabolism may act in combination to associate with the risk of cancer. At advanced stages, more cancer cells may encounter elevated metabolic stresses, and more nutrient-available cells can secrete homocysteine to avoid homocysteine toxicity and maintain cellular homocysteine homeostasis for those more metabolically stressed cancer cells. Such “cross-feeding” could be common among metabolically heterogeneous cancer cells. In this capacity, therefore, any role of homocysteine metabolism is likely to be complex, possibly involving a dual cancer-initiate effect in cooperation with other risk factors.

Mendelian randomization can utilize genetic instruments to analyze a large number of patient samples because of increasingly/readily more available published data from diverse GWAS datasets, which can significantly increase the accuracy thus the credibility of analyses. To the best of our knowledge, this is the first two-sample MR study that has accurately evaluated potential associations between plasma homocysteine level and cancer risk. The advantage of this MR approach is that genetic variation is a long-term and stable cause of exposure. Compared with randomized controlled trials, it is elaborated directly and is not subject to interferences by confound factors like social environment and lifestyle. In this particular study, the LD analyses were used to rule out three SNPs, and the MR-Egger intercept test and sensitivity analyses showed no pleiotropic effect of the 15 selected SNPs, which additionally increases the credibility of our study.

However, our study is not without limitations. The data that the MR analyses utilized have been collected from earlier published available GWAS summary data that have been adjusted. Further, the grouped data did not allow us to effectively explore the associations between homocysteine levels with different stages of cancer and different hormone status of different cancers. In a broader sense, the methionine cycle is but one of the pathways of one-carbon metabolism; further studies are needed to explore whether other biochemical components involved in one-carbon-fixation pathways, such as those engaged in *de novo* nucleotide biosynthetic pathways, are associated with risks of cancer development.

## Conclusions

In conclusion, our study demonstrated no evidence of causal associations between plasma homocysteine and cancers of the BRCA, PrCa, and RCC via a two-sample MR approach. Herein, our MR study suggested that homocysteine alone might not be useful as a dynamic biomarker alone for the risks of the aforementioned three cancers.

## Data Availability Statement

The original contributions presented in the study are included in the article/Supplementary Material, further inquiries can be directed to the corresponding author/s.

## Author Contributions

QH, YS, and YaL conceived the study design and drafted the manuscript. ZY, ZQ and JL participated in data extraction and data analysis. XJ and YiL did the data checking and analysis. All authors critically reviewed the manuscript.

## Conflict of Interest

The authors declare that the research was conducted in the absence of any commercial or financial relationships that could be construed as a potential conflict of interest.

## Publisher's Note

All claims expressed in this article are solely those of the authors and do not necessarily represent those of their affiliated organizations, or those of the publisher, the editors and the reviewers. Any product that may be evaluated in this article, or claim that may be made by its manufacturer, is not guaranteed or endorsed by the publisher.

## Abbreviations

BRCA: breast cancer
PrCa: prostate cancer
MR: mendelian randomization
RCC: renal cell carcinoma
SNP: single-nucleotide polymorphism
GWAS: genome-wide association studies
IVW: inverse-variance-weighted
IV: instrumental variable
LD: linkage disequilibrium
MAF: minor allele frequency
HWE: Hardy-Weinberg equilibrium
TUK1&TUK2: the twinsUK cohort
WGHS: women's genome health study
NHS: nurses' health study.

## References

[1] Eriksson S Prigge JR Talago EA Arner ES Schmidt EE. Dietary methionine can sustain cytosolic redox homeostasis in the mouse liver. Nat Commun. (2015). 6:6479. 10.1038/ncomms7479 PMC4369796 25790857

[2] Ducker GS Rabinowitz JD. One-carbon metabolism in health and disease. Cell Metab. (2017). 25:27–42. 10.1016/j.cmet.2016.08.009 27641100 PMC5353360

[3] Mudd SH Brosnan JT Brosnan ME Jacobs RL Stabler SP Allen RH. Methyl balance and transmethylation fluxes in humans. Am J Clin Nutr. (2007). 85:19–25. 10.1093/ajcn/85.1.19 17209172

[4] Pajares MA Perez-Sala D. Betaine homocysteine S-methyltransferase: just a regulator of homocysteine metabolism? Cell Mol Life Sci. (2006). 63:2792–803. 10.1007/s00018-006-6249-6 17086380 PMC11136095

[5] Perla-Kajan J Twardowski T Jakubowski H. Mechanisms of homocysteine toxicity in humans. Amino Acids. (2007). 32:561–72. 10.1007/s00726-006-0432-9 17285228

[6] McNulty H Scott JM. Intake and status of folate and related B-vitamins: considerations and challenges in achieving optimal status. Br J Nutr. (2008). 99(Suppl 3):S48–54. 10.1017/S0007114508006855 18598588

[7] Casas JP Bautista LE Smeeth L Sharma P Hingorani AD. Homocysteine and stroke: evidence on a causal link from Mendelian randomisation. Lancet. (2005). 365:224–32. 10.1016/S0140-6736(05)70152-5 15652605

[8] Wald DS Law M Morris JK. Homocysteine and cardiovascular disease: evidence on causality from a meta-analysis. BMJ. (2002). 325:1202. 10.1136/bmj.325.7374.1202 PMC135491 12446535

[9] Holmes MV Newcombe P Hubacek JA Sofat R Ricketts SL Cooper J. Effect modification by population dietary folate on the association between MTHFR genotype, homocysteine, and stroke risk: a meta-analysis of genetic studies and randomised trials. Lancet. (2011). 378:584–94. 10.1016/S0140-6736(11)60872-6 21803414 PMC3156981

[10] Bicikova M Hampl R Hill M Stanicka S Tallova J Vondra K. Steroids, sex hormone-binding globulin, homocysteine, selected hormones and markers of lipid and carbohydrate metabolism in patients with severe hypothyroidism and their changes following thyroid hormone supplementation. Clin Chem Lab Med. (2003). 41:284–92. 10.1515/CCLM.2003.045 12705335

[11] Guimarães DA Cardoso J Dusse LM Franco RM Franco Hde A Alvim TC. Effect of oral hormone replacement therapy on plasma homocysteine levels. Acta Obstet Gynecol Scand. (2006). 85:1304–6. 10.1080/00016340600676136 17091407

[12] Sun CF Haven TR Wu TL Tsao KC Wu JT. Serum total homocysteine increases with the rapid proliferation rate of tumor cells and decline upon cell death: a potential new tumor marker. Clin Chim Acta. (2002). 321:55–62. 10.1016/S0009-8981(02)00092-X 12031593

[13] Wu LL Wu JT. Hyperhomocysteinemia is a risk factor for cancer and a new potential tumor marker. Clin Chim Acta. (2002). 322:21–8. 10.1016/S0009-8981(02)00174-2 12104077

[14] Ames BN. DNA damage from micronutrient deficiencies is likely to be a major cause of cancer. Mutat Res. (2001). 475:7–20. 10.1016/S0027-5107(01)00070-7 11295149

[15] Duthie SJ Narayanan S Brand GM Pirie L Grant G. Impact of folate deficiency on DNA stability. J Nutr. (2002). 132(8 Suppl):2444S−9S. 10.1093/jn/132.8.2444S 12163709

[16] Lin J Lee IM Song Y Cook NR Selhub J Manson JE. Plasma homocysteine and cysteine and risk of breast cancer in women. Cancer Res. (2010). 70:2397–405. 10.1158/0008-5472.CAN-09-3648 20197471 PMC2840179

[17] Gatt A Makris A Cladd H Burcombe RJ Smith JM Cooper P. Hyperhomocysteinemia in women with advanced breast cancer. Int J Lab Hematol. (2007). 29:421–5. 10.1111/j.1751-553X.2007.00907.x 17988296

[18] Chou YC Lee MS Wu MH Shih HL Yang T Yu CP. Plasma homocysteine as a metabolic risk factor for breast cancer: findings from a case-control study in Taiwan. Breast Cancer Res Treat. (2007). 101:199–205. 10.1007/s10549-006-9278-9 16850249

[19] Wu X Zou T Cao N Ni J Xu W Zhou T. Plasma homocysteine levels and genetic polymorphisms in folate metablism are associated with breast cancer risk in chinese women. Hered Cancer Clin Pract. (2014). 12:2. 10.1186/1897-4287-12-2 PMC3936891 24559276

[20] Gohlke JH Lloyd SM Basu S Putluri V Vareed SK Rasaily U. Methionine-homocysteine pathway in African-American prostate cancer. JNCI Cancer Spectr. (2019). 3:pkz019. 10.1093/jncics/pkz019 PMC6489686 31360899

[21] Wu K Helzlsouer KJ Comstock GW Hoffman SC Nadeau MR Selhub J. A prospective study on folate, B12, and pyridoxal 5′-phosphate (B6) and breast cancer. Cancer Epidemiol Biomarkers Prev. (1999). 8:209–17. 10090298

[22] Zhang SM Willett WC Selhub J Hunter DJ Giovannucci EL Holmes MD. Plasma folate, vitamin B6, vitamin B12, homocysteine, and risk of breast cancer. J Natl Cancer Inst. (2003). 95:373–80. 10.1093/jnci/95.5.373 12618502

[23] Agnoli C Grioni S Krogh V Pala V Allione A Matullo G. Plasma riboflavin and vitamin B-6, but not homocysteine, folate, or vitamin b-12, are inversely associated with breast cancer risk in the European prospective investigation into cancer and nutrition-varese cohort. J Nutr. (2016). 146:1227–34. 10.3945/jn.115.225433 27121532

[24] Collin SM Metcalfe C Refsum H Lewis SJ Zuccolo L Smith GD. Circulating folate, vitamin B12, homocysteine, vitamin B12 transport proteins, and risk of prostate cancer: a case-control study, systematic review, and meta-analysis. Cancer Epidemiol Biomarkers Prev. (2010). 19:1632–42. 10.1158/1055-9965.EPI-10-0180 20501771 PMC3759018

[25] Bray F Ferlay J Soerjomataram I Siegel RL Torre LA Jemal A. Global cancer statistics 2018: GLOBOCAN estimates of incidence and mortality worldwide for 36 cancers in 185 countries. CA Cancer J Clin. (2018). 68:394–424. 10.3322/caac.21492 30207593

[26] Crandall CJ Aragaki AK Cauley JA McTiernan A Manson JE Anderson G. Breast tenderness and breast cancer risk in the estrogen plus progestin and estrogen-alone women's health initiative clinical trials. Breast Cancer Res Treat. (2012). 132:275-85. 10.1007/s10549-011-1848-9 PMC3697872 22042371

[27] Yager JD Davidson NE. Estrogen carcinogenesis in breast cancer. N Engl J Med. (2006). 354:270–82. 10.1056/NEJMra050776 16421368

[28] Perou CM Sorlie T Eisen MB van de Rijn M Jeffrey SS Rees CA. Molecular portraits of human breast tumours. Nature. (2000). 406:747–52. 10.1038/35021093 10963602

[29] Iqbal J Kahane A Park AL Huang T Meschino WS Ray JG. Hormone levels in pregnancy and subsequent risk of maternal breast and ovarian cancer: a systematic review. J Obstet Gynaecol Can. (2019). 41:217–22. 10.1016/j.jogc.2018.03.133 30528445

[30] Cuzick J Thorat MA Andriole G Brawley OW Brown PH Culig Z. Prevention and early detection of prostate cancer. Lancet Oncol. (2014). 15:e484–92. 10.1016/S1470-2045(14)70211-6 25281467 PMC4203149

[31] Wang G Zhao D Spring DJ DePinho RA. Genetics and biology of prostate cancer. Genes Dev. (2018). 32:1105–40. 10.1101/gad.315739.118 30181359 PMC6120714

[32] Hammerer P Madersbacher S. Landmarks in hormonal therapy for prostate cancer. BJU Int. (2012). 110(Suppl 1):23–9. 10.1111/j.1464-410X.2012.11431.x 23046037

[33] Dunzendorfer U Drahovsky D Schmidt-Gayk H. [Peptide hormones LH, FSH, TSH, prolactin, beta-HCG and PTH in patients with urogenital tumors]. Onkologie. (1981). 4:188–92. 10.1159/000214908 6795555

[34] Zucchetto A Talamini R Dal Maso L Negri E Polesel J Ramazzotti V. Reproductive, menstrual, and other hormone-related factors and risk of renal cell cancer. Int J Cancer. (2008). 123:2213–6. 10.1002/ijc.23750 18711701

[35] Concolino G Marocchi A Conti C Tenaglia R Di Silverio F Bracci U. Human renal cell carcinoma as a hormone-dependent tumor. Cancer Res. (1978). 38(11 Pt 2):4340–4. 698974

[36] Latvala A Ollikainen M. Mendelian randomization in (epi)genetic epidemiology: an effective tool to be handled with care. Genome Biol. (2016). 17:156. 10.1186/s13059-016-1018-9 PMC4944517 27418254

[37] Gala H Tomlinson I. The use of Mendelian randomisation to identify causal cancer risk factors: promise and limitations. J Pathol. (2020). 250:541–54. 10.1002/path.5421 32154591

[38] van Meurs JB Pare G Schwartz SM Hazra A Tanaka T Vermeulen SH. Common genetic loci influencing plasma homocysteine concentrations and their effect on risk of coronary artery disease. Am J Clin Nutr. (2013). 98:668–76. 10.3945/ajcn.112.044545 23824729 PMC4321227

[39] Zhang H Ahearn TU Lecarpentier J Barnes D Beesley J Qi G. Genome-wide association study identifies 32 novel breast cancer susceptibility loci from overall and subtype-specific analyses. Nat Genet. (2020). 52:572–81. 10.1038/s41588-020-0609-2 32424353 PMC7808397

[40] Schumacher FR Al Olama AA Berndt SI Benlloch S Ahmed M Saunders EJ. Association analyses of more than 140,000 men identify 63 new prostate cancer susceptibility loci. Nat Genet. (2018). 50:928–36. 10.1038/s41588-018-0142-8 29892016 PMC6568012

[41] Laskar RS Muller DC Li P Machiela MJ Ye Y Gaborieau V. Sex specific associations in genome wide association analysis of renal cell carcinoma. Eur J Hum Genet. (2019). 27:1589–98. 10.1038/s41431-019-0455-9 31231134 PMC6777615

[42] Burgess S Butterworth A Thompson SG. Mendelian randomization analysis with multiple genetic variants using summarized data. Genet Epidemiol. (2013). 37:658–65. 10.1002/gepi.21758 24114802 PMC4377079

[43] Bowden J Davey Smith G Haycock PC Burgess S. Consistent estimation in Mendelian randomization with some invalid instruments using a weighted median estimator. Genet Epidemiol. (2016). 40:304–14. 10.1002/gepi.21965 27061298 PMC4849733

[44] Love RR Anker G Yang Y Refsum H Ueland PM Lonning PE. Serum homocysteine levels in postmenopausal breast cancer patients treated with tamoxifen. Cancer Lett. (1999). 145:73–7. 10.1016/S0304-3835(99)00233-5 10530772

[45] Stathopoulou A Vlachonikolis I Mavroudis D Perraki M Kouroussis C Apostolaki S. Molecular detection of cytokeratin-19-positive cells in the peripheral blood of patients with operable breast cancer: evaluation of their prognostic significance. J Clin Oncol. (2002). 20:3404–12. 10.1200/JCO.2002.08.135 12177100

[46] Rickles FR Levine M Edwards RL. Hemostatic alterations in cancer patients. Cancer Metastasis Rev. (1992). 11:237–48. 10.1007/BF01307180 1423816

