# Supplementary material for: The Impact of Homocysteine on the Risk of Hormone-Related Cancers: A Mendelian Randomization Study
Source: Front Nutr. 2021 Aug 24;8:645371. doi: 10.3389/fnut.2021.645371 (PMC8421785; doi:10.3389/fnut.2021.645371)
Supplement: Supplementary file 1 [file Data_Sheet_1.docx]

**Supplementary Material**

Table S1. Calculation of linkage disequilibrium of selected SNPs

Table S1A. Calculation of linkage disequilibrium of 4 selected SNPs on Chr.1

| RS_number | rs12134663 | rs1801133 | rs4660306 | rs2275565 |
| --- | --- | --- | --- | --- |
| rs12134663 | 1 | 0.094 | 0.012 | 0.002 |
| rs1801133 | 0.094 | 1 | 0.015 | 0.011 |
| rs4660306 | 0.012 | 0.015 | 1 | 0.001 |
| rs2275565 | 0.002 | 0.011 | 0.001 | 1 |

Table S1B. Calculation of linkage disequilibrium of 2 selected SNPs on Chr.6

| RS_number | rs548987 | rs9369898 |
| --- | --- | --- |
| rs548987 | 1 | 0.003 |
| rs9369898 | 0.003 | 1 |

Table S1C. Calculation of linkage disequilibrium of 2 selected SNPs on Chr.10

| RS_number | rs1801222 | rs12780845 |
| --- | --- | --- |
| rs1801222 | 1 | 0.007 |
| rs12780845 | 0.007 | 1 |

Table S1D. Calculation of linkage disequilibrium of 2 selected SNPs on Chr.11

| RS_number | rs7130284 | rs957140 |
| --- | --- | --- |
| rs7130284 | 1 | 0.05 |
| rs957140 | 0.05 | 1 |

Table S1E. Calculation of linkage disequilibrium of 2 selected SNPs on Chr.16

| RS_number | rs154657 | rs12921383 |
| --- | --- | --- |
| rs154657 | 1 | 0.111 |
| rs12921383 | 0.111 | 1 |

Table S1F. Calculation of linkage disequilibrium of 2 selected SNPs on Chr.21

| RS_number | rs234709 | rs2851391 |
| --- | --- | --- |
| rs234709 | 1 | 0.006 |
| rs2851391 | 0.006 | 1 |

Population = (CEU) Utah Residents from North and West Europe; r^2^ < 0.05.

Table S2. Related traits of homocysteine-associated SNPs.

| SNP | Chromosome Position | Nearby Gene | Trait | β | P value |
| --- | --- | --- | --- | --- | --- |
| rs2275565 | 1：235115299 | MTR | Homocysteine levels | -0.0542 | 1.96E-43 |
| rs4660306 | 1：45751262 | MMACHC | Homocysteine levels | 0.0435 | 2.33E-12 |
| rs12134663 | 1:11778589 | MTHFR | Homocysteine levels | -0.101 | 2.54E-09 |
| rs1047891 | 2:210675783 | CPS1 | Chronic kidney disease, High density lipoprotein,HDL cholesterol, Homocysteine levels | 0.0864 | 4.58E-27 |
| rs9369898 | 6:49382193 | MUT | Homocysteine levels | 0.0449 | 2.17E-24 |
| rs548987 | 6:25869371 | SLC17A3 | Homocysteine levels | 0.0597 | 1.12E-11 |
| rs42648 | 7：89815696 | GTPB10 | Homocysteine levels | -0.0395 | 1.97E-10 |
| rs1801222 | 10：17196157 | CUBN | Folate pathway vitamin levels, Homocysteine levels, Vitamin B levels in ischemic stroke | 0.0453 | 8.43E-10 |
| rs12780845 | 10:17223244 | CUBN | Homocysteine levels | 0.0529 | 7.8E-09 |
| rs7130284 | 11: 88788020 | NOX4 | Homocysteine levels, Vitamin and mineral supplements: multivitamins + or - minerals | -0.1242 | 1.88E-21 |
| rs2251468 | 12：119889509 | HNF1A | Homocysteine levels, Total cholesterol | -0.0512 | 1.28E-10 |
| rs154657 | 16：88235597 | DPEP1 | Homocysteine levels | 0.0963 | 1.74E-20 |
| rs838133 | 19：53951341 | FUT2 | Homocysteine levels | 0.0422 | 7.48E-10 |
| rs234709 | 21：43360033 | CBS | Homocysteine levels | -0.0718 | 3.9E-12 |
| rs2851391 | 21:44487404 | CBS | Blood metabolite levels, Homocysteine levels, Obesity | 0.056 | 1.7E-08 |

The included traits were found to be related with 15 homocysteine-associated SNPs and were thought to be associated with all the outcomes. The results were obtained from PhenoScanner V2 website (http://www.phenoscan ner.medschl.cam.ac.uk).

Table S3. The association information of homocysteine-associated SNPs with breast cancer in all participants

| SNP | EA | Overall breast cancer | |
| --- | --- | --- | --- |
|  |  | β(SE) | P value |
| rs2275565 | T | 0.0032 (0.0073) | 0.6647 |
| rs4660306 | T | -0.0077 (0.0063) | 0.2216 |
| rs12134663 | A | 0.0139 (0.0080) | 0.0804 |
| rs1047891 | A | 0.0098 (0.0067) | 0.1451 |
| rs9369898 | A | -0.0016 (0.0062) | 0.7918 |
| rs548987 | C | -0.0343 (0.0095) | 0.0003 |
| rs42648 | A | 0.0013 (0.0061) | 0.8360 |
| rs1801222 | A | 0.0140 (0.0063) | 0.0262 |
| rs12780845 | A | -0.0019 (0.0065) | 0.7718 |
| rs7130284 | T | -0.0083 (0.0116) | 0.4766 |
| rs2251468 | A | 0.0189 (0.0063) | 0.0026 |
| rs154657 | A | -0.0068 (0.0061) | 0.2681 |
| rs838133 | A | -0.0048 (0.0064) | 0.4466 |
| rs234709 | T | -0.0031 (0.0061) | 0.6126 |
| rs2851391 | T | -0.0132 (0.0060) | 0.0285 |

EA = effect allele; β = per allele effect on SD units; SE = standard error; P value = p-value for the genetic association.

Table S4. The association information of homocysteine-associated SNPs with prostate cancer in all participants

| SNP | EA | prostate cancer | |
| --- | --- | --- | --- |
|  |  | β(SE) | P value |
| rs2275565 | T | -0.0098 (0.0097) | 0.3133 |
| rs4660306 | T | 0.0176 (0.0084) | 0.03584 |
| rs12134663 | A | 0.0089 (0.0107) | 0.409 |
| rs1047891 | A | 0.0123 (0.009) | 0.1709 |
| rs9369898 | A | 0.0013 (0.0083) | 0.8707 |
| rs548987 | C | -0.0298 (0.0121) | 0.0138 |
| rs42648 | A | 0.0167 (0.0082) | 0.0428 |
| rs1801222 | A | -0.0089 (0.0085) | 0.2958 |
| rs12780845 | A | 0.0014 (0.0087) | 0.8698 |
| rs7130284 | T | 0.0002 (0.0152) | 0.9916 |
| rs2251468 | A | -0.0109 (0.0084) | 0.193 |
| rs154657 | A | 0.0009 (0.0082) | 0.908 |
| rs838133 | A | 0.0083 (0.0085) | 0.3304 |
| rs234709 | T | 0.0017 (0.0081) | 0.8315 |
| rs2851391 | T | 0.0022 (0.0081) | 0.7891 |

EA = effect allele; β = per allele effect on SD units; SE = standard error; P value = p-value for the genetic association.

Table S5. The association information of homocysteine-associated SNPs with RCC in all participants and single sex

| SNP | EA | renal cell carcinoma in women | | renal cell carcinoma in men | |
| --- | --- | --- | --- | --- | --- |
|  |  | β(SE) | P value | β(SE) | P value |
| rs2275565 | T | -0.0692 (0.0507) | 0.17 | -0.0346 (0.0411) | 0.4 |
| rs4660306 | T | -0.0111 (0.0455) | 0.81 | -0.0368 (0.0363) | 0.31 |
| rs12134663 | A | 0.0284 (0.056) | 0.61 | -0.034 (0.0449) | 0.45 |
| rs1047891 | A | 0.0511 (0.05) | 0.31 | 0.029 (0.0398) | 0.47 |
| rs9369898 | A | 0.0019 (0.0443) | 0.97 | 0.0244 (0.0361) | 0.5 |
| rs548987 | C | -0.061 (0.06692) | 0.38 | -0.0356 (0.0554) | 0.52 |
| rs42648 | A | 0.0399 (0.0448) | 0.37 | -0.0512 (0.0361) | 0.16 |
| rs1801222 | A | 0.0363 (0.0461) | 0.43 | 0.0116 (0.0372) | 0.76 |
| rs12780845 | A | 0.0258 (0.0466) | 0.58 | 0.0213 (0.0371) | 0.57 |
| rs7130284 | T | 0.0847 (0.0784) | 0.28 | -0.049 (0.0656) | 0.46 |
| rs2251468 | A | -0.0682 (0.0445) | 0.13 | 0.0016 (0.0359) | 0.97 |
| rs154657 | A | -0.0939 (0.0447) | 0.04 | -0.0082 (0.036) | 0.82 |
| rs838133 | A | -0.0239 (0.0492) | 0.63 | -0.0213 (0.0394) | 0.59 |
| rs234709 | T | 0.0201 (0.0443) | 0.65 | 0.026 (0.0354) | 0.46 |
| rs2851391 | T | 0.0226 (0.0438) | 0.61 | 0.0269 (0.0347) | 0.44 |

EA = effect allele; β = per allele effect on SD units; SE = standard error; P value = p-value for the genetic association.

Table S6. IVW analysis for genetic associations between plasma homocysteine levels and all the outcomes

| Outcome | Effect (95% CI) | P value |
| --- | --- | --- |
| Overall BRCA | 0.97(0.90, 1.06) | 0.543 |
|  |  |  |
|  |  |  |
| Overall PrCa | 1.01 (0.93, 1.11) | 0.774 |
| RCC in women | 0.99 (0.73, 1.34) | 0.929 |
| RCC in men | 0.89 (0.61, 1.31) | 0.563 |

Effect = odds ratio of the estimates of causal associations between homocysteine levels and outcomes; CI = confidence interval; P value = p-value of the causal estimate.

Table S7. Weighted median and MR-Egger analysis for genetic associations between plasma homocysteine levels and all the outcomes

| Method | Weighted median | MR-Egger | |
| --- | --- | --- | --- |
|  |  | Estimate | Intercept |
| Overall BRCA |  |  |  |
| Estimate (95% CI) | -0.012 (-0.086, 0.062) | 0.109 (-0.115, 0.333) | -0.010 (0.006, 0.207) |
| P value | 0.757 | 0.341 | 0.207 |
|  |  |  |  |
|  |  |  |  |
|  |  |  |  |
|  |  |  |  |
|  |  |  |  |
|  |  |  |  |
| Overall PrCa |  |  |  |
| Estimate (95% CI) | 0.011 (-0.088, 0.110) | -0.01 (-0.285, 0.264) | 0.002 (-0.016, 0.019) |
| P value | 0.83 | 0.941 | 0.860 |
| RCC in women |  |  |  |
| Estimate (95% CI) | -0.280 (-0.824, 0.264) | -0.911 (-2.046, 0.233) | 0.055 (-0.019, 0.128) |
| P value | 0.582 | 0.115 | 0.143 |
| RCC in men |  |  |  |
| Estimate (95% CI) | -0.115 (-0.526, 0.296) | -0.384 (-1.303, 0.535) | 0.025 (-0.034, 0.085) |
| P value | 0.313 | 0.412 | 0.402 |
